# From Petri Dishes to Organ on Chip Platform: The Increasing Importance of Machine Learning and Image Analysis

**DOI:** 10.3389/fphar.2019.00100

**Published:** 2019-02-26

**Authors:** Arianna Mencattini, Fabrizio Mattei, Giovanna Schiavoni, Annamaria Gerardino, Luca Businaro, Corrado Di Natale, Eugenio Martinelli

**Affiliations:** ^1^Department of Electronic Engineering, University of Rome Tor Vergata, Rome, Italy; ^2^Department of Oncology and Molecular Medicine, Istituto Superiore di Sanità, Rome, Italy; ^3^CNR, Institute for Photonics and Nanotechnologies, Rome, Italy

**Keywords:** organ on chip, time-lapse microscopy, machine learning, image analysis, cell interaction analysis

## Abstract

The increasing interest for microfluidic devices in medicine and biology has opened the way to new time-lapse microscopy era where the amount of images and their acquisition time will become crucial. In this optic, new data analysis algorithms have to be developed in order to extract novel features of cell behavior and cell–cell interactions. In this brief article, we emphasize the potential strength of a new paradigm arising in the integration of microfluidic devices (i.e., organ on chip), time-lapse microscopy analysis, and machine learning approaches. Some snapshots of previous case studies in the context of immunotherapy are included as proof of concepts of the proposed strategies while a visionary description concludes the work foreseeing future research and applicative scenarios.

With its invention in 1590, microscopy abruptly gave us access to a completely new world. It uses radiation and a system of lenses to study processes and structures at the micro scale and below. Optical microscopy is used extensively in microelectronics, physics, biotechnology, pharmaceutical research, cell macrostructure investigations and microbiology and has been demonstrated a fundamental imaging technique for modern cell biology and immune-oncology research where dealing with free cells or tissue fragments. The advent of semiconductor-based systems and modern developments in Complementary Metal-Oxide Semiconductor (CMOS) and charge-coupled device (CCD) cameras allowed changing the final perspective providing the capability to pass from optical images to matrices of numbers (Thorn, 2016). In such scenario, computer algorithms developed to process digital objects have become a fundamental tool to increase the precision of investigation and the amount of information extracted from each experiment. However, what is really limiting the strength of analysis is the difficulty in aggregating expertise from different areas, i.e., biological fields as well image analysis, pattern recognition, technology, and sensor devices. Such convergence allows going further in the biological studies, passing from static visualization to the real-time analysis of cells, through the acquisition of video sequences (Figure 1) with the intent to study its evolution under controlled experimental conditions (Chen et al., 2006). Moreover, focus on cell motility has not yet been fully addressed until now. As well established, cell migration is a fundamental process for life (Li et al., 2017) being involved in bacteria collective motion, in the morphogenesis of pluricellular organisms, in adult physiological process (such as tissue repair and immune cell trafficking), and in many cancer-related diseases (such as cancer metastasis) (Gupta and Massagué, 2006) and immunotherapies (Nguyen et al., 2018). The large variety of biological compartments involved in cell-motility makes it striking to reconstitute the environment in microfluidic devices in order to analyze the collective behavior of moving cells minimizing the effects of external conditions (Sackmann et al., 2014). The Organ-on-chip (OOC) approach expands the traditional concept of standard cell culture methods, offering the opportunity to co-culture a large number of trackable cell types, in a variety of 2D and 3D microenvironments (Huh et al., 2010; Businaro et al., 2013; Bhatia and Ingber, 2014). OOC technology development has the aim to replicate diverse organ functionalities with *in vitro* models (Kamm et al., 2018). However, while the OOC technologies are still at a relatively early stage in development, nascent versions of cardiac muscle (Lind et al., 2017), liver (Domansky et al., 2010), brain (Adriani et al., 2017), lung (Huh et al., 2010), skin (Mori et al., 2017), placenta (Miura et al., 2015) have been reported. Similar systems have been designed to provide new insights into fundamental disease processes such as cancer (Chen et al., 2016) and Alzheimer’s disease (Choi et al., 2013). Practical challenges of OOCs may include phenotypic instability, low throughput associated with system complexity, material-drug incompatibilities of commonly used device materials such as PDMS, and biomaterial inconsistencies and limitations. A fundamental question for OOC technology is if it will be able to create microscale constructs that adequately recapitulate the macroscopic organs. Two major scaling issues arise in OOC design and construction on the ability to maintain absolute values of physiological parameters and relative sizes between different types of cells, tissues, and organs. For all these reasons, a massive analysis through the use of video processing and machine learning is even more required in order to demonstrate the validity of OOC solutions and their actual capability to be ready for being embedded into a network of OOCs toward a more complex and realistic *in vivo* like environment.

**FIGURE 1:**
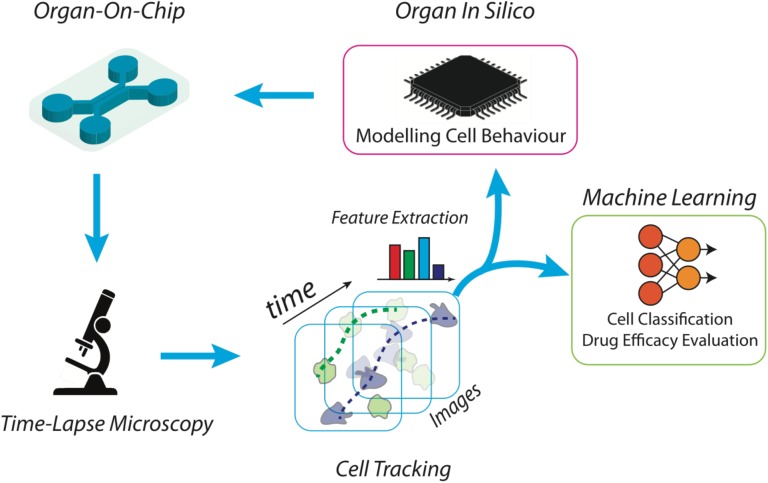
Scheme of a high throughput platform for the advanced study and reproduction of the tumor microenvironment. The microfluidic device is manufactured *ad hoc*, according to the biological experiment requirements. Then, the desired cell subsets are loaded into the chip together with tumor cells, to an extent to propose a simplified version of the tumor microenvironment. Time-lapse microscopy is used to acquire the high-resolution frames of the whole video sequence. Microscopy setting is functionalized by the scale of the objects of interest and the duration of the time-lapse. Cells are then automatically localized and tracked across each frame of the video sequence and trajectories are characterized in terms of individual and aggregated kinematics and morphological descriptors. At this point, specifically developed machine learning algorithms are then applied to recognize patterns for biological reasoning. For example, cell tracking datasets are then clustered into separated groups reflecting distinct cell behaviors. The same kinematics and morphological descriptors can be used as input for *in silico* models aimed at simulating on-chip experiments.

In this context, one of the most challenging scenarios for OOC devices is represented by cancer-immune cross-talk due to the very complex and not still completely discovered signaling modalities between immune cells and cancer insult or among clustered cancer cells. Some attempts have been presented with the aim to model cancer–immune interaction (Vacchelli et al., 2015) through time-lapse microscopy analysis. Critical is the need to translate moving cells into trajectories, and kinematics descriptors using label-free artificial intelligence architecture. Machine learning becomes hence a key component of such a virtual laboratory to manage and analyze a large amount of data describing biological complexity and introduces to the new definition of the so called *in silico* experiments. First, cells should be located and tracked through the video sequence by means of automatic cell tracking software [an example can be Cell Hunter approach (Biselli et al., 2017; Parlato et al., 2017; Figure 1) but there are several open software’s in the literature (Chenouard et al., 2014)]. In this field, challenging aspects are the need to locate cells using non-invasive strategies and to reliably track cells in highly dense heterogeneous cultures (Chenouard et al., 2014; Biselli et al., 2017; Parlato et al., 2017). Trajectories of moving cells have to be then translated into kinematics descriptors (such as speed, angular direction, persistence, directionality, step length, etc.) and, in presence of clustered cells (Di Giuseppe et al., 2019), automatic cell clustering has to be performed in order to aggregate cells exhibiting a similar kinematic activity. In this regard, it is of key note to mention that a group of cells apparently different from a biological point of view can be clustered by using the aforementioned parameters, such as cells having the same step length. As a mere example, it has been demonstrated that Formyl Receptor 1 (FPR1) expression in peripheral blood monocytes can modulate the extent of their step length (Biselli et al., 2017). After cell motions has been quantified in terms of numerical features, machine learning algorithms [Deep Learning, Support Vector Machine, Discriminant Analysis, etc. (Jordan and Mitchell, 2015)] can be then exploited to recognize common patterns (e.g., target direction, motion kinds) in different cells and/or cell clusters in order to understand the biological behavior with respect to contaminants, insults, chemical stimuli, etc. (Figure 1). The study of tumor microenvironment represents an application scenario where this approach will have a tremendous impact in the near future. This is a complex biological entity composed of different cell types, e.g., cancer cells, fibroblasts, pericytes and immune cells, whose mutual interaction dictates cancer progression and metastatic spread. The application of OOC in the context of the tumor microenvironment allows to separately study the migratory behavior of specific immune cell subsets toward the tumor cells loaded in a separate chamber (e.g., dendritic cells, T lymphocytes). For example, cancer cell movements alone can be analyzed to discover cancer leadership, invasion and segregation phenomena (Kabla, 2012). Additional applicative scenarios include tumor cells such as murine fibrosarcoma cell line loaded together with spleen cells in which DC (an immune cell subset) are labeled (and thus identified) with a DC-specific antibody such as anti-CD11c antibody. In these experimental settings, DCs are monitored on-chip in presence of tumor cells by yielding the aforementioned cell tracking parameter by Cell Hunter method (Nguyen et al., 2018). Motility studies are fundamental not only as further proof of concept of elsewhere verified therapeutic effects but also to discover unexpected cell behavior. As an example, cancer cells may behave differently according to the position in the cell cluster and to the motion of neighboring cells. In this way, quantifying cells motility may also reveal cancer mechanisms in support of future treatments (Yasuda, 2013; Klemm and Joyce, 2015). Furthermore, the prediction capability of such a platform for analysis may allow not only to understand what is under the microscope but definitely to serve as feedback for further OOCs experiments. For example, modeling coordinated cell movements (Comes et al., 2019) allow reconstituting totally *in silico* artificial videos of cell movements. Video analysis and machine learning algorithms are then tuned according to the synthetic videos in which the user may also simulate the presence of drugs through specific mathematical equations. In this way, dynamic drug administration may be performed (e.g., precise modulation of drug concentration) according to cell movements extracted in synthetic videos with the effect to make the *ex vivo* experiment more robust, to conduct synthetic massive tests, to reduce time-to therapy and patient undesired treatment effects.

The described platform is expected to open new scenarios of cell network modeling such as agent-based system (e.g., autonomous model of each cell within an interacting network of agents) in order to simulate more advanced organs-on-chip model coupled to artificial intelligence (AI)-based algorithms and neural networks to provide more affordable organs-on-chip models of study. These AI-based OOC may potentially shorten the distances between *in vivo* and *ex vivo* like scenarios in favor of reduced invasiveness and deep understanding.

Future application scenarios include the possibility to develop a hybrid system in which OOC-based environment interacts with *in silico*-based devices. Such a scenario allows enlarging the potentiality of OOCs experiments by increasing the mimicking capability of the platform toward *in vivo* like environment.

This scenario is made possible counteracting the limitations imposed by the need of very high frame rate and high spatial resolution of the acquired images that lead to undesired effects such as phototoxicity and storage of more than Gigabytes of a single experimental data. Thanks to the exploitation of modern Deep Learning (DL) architecture (Goodfellow et al., 2014), future hybrid OOCs device could adopt DL strategies to virtually increase the video quality thus maintaining accurate analysis results reducing cell illumination rates and memory storage and increasing at the same time the capability to follow the system evolution.

## Author Contributions

EM and AM designed the manuscript organization and coordinated interactions among authors. All authors contributed to the manuscript preparation and revision.

## Conflict of Interest Statement

The authors declare that the research was conducted in the absence of any commercial or financial relationships that could be construed as a potential conflict of interest.
